# Maximum Entropy Theory of Ecology: A Reply to Harte

**DOI:** 10.3390/e20050308

**Published:** 2018-04-24

**Authors:** Marco Favretti

**Affiliations:** Dipartimento di Matematica “Tullio Levi-Civita”, Università degli Studi di Padova, 35122 Padova, Italy; favretti@math.unipd.it

**Keywords:** Maximum Entropy principle, Maximum Entropy Theory of Ecology, Shannon Entropy, Boltzmann counting

## Abstract

In a paper published in this journal, I addressed the following problem: under which conditions will two scientists, observing the same system and sharing the same initial information, reach the same probabilistic description upon the application of the Maximum Entropy inference principle (MaxEnt) independent of the probability distribution chosen to set up the MaxEnt procedure. This is a minimal objectivity requirement which is generally asked for scientific investigation. In the same paper, I applied the findings to a critical examination of the application of MaxEnt made in Harte’s Maximum Entropy Theory of Ecology (METE). Prof. Harte published a comment to my paper and this is my reply. For the sake of the reader who may be unaware of the content of the papers, I have tried to make this reply self-contained and to skip technical details. However, I invite the interested reader to consult the previously published papers.

## 1. On the Application of the Maximum Entropy Principle

I thank Prof. Harte for the attention devoted to my paper. The applications of Jaynes’ Maximum Entropy Principle (MaxEnt, [1]) are among my research interests and I am happy to say that the inspiration for writing the paper [2] commented by Harte came from a sustained effort to penetrate the foundational aspects of Maximum Entropy Theory of Ecology (METE) theory.

Before giving a brief description of METE in Section 2, I would like to warn the reader that in [2] I am not constructing a different theory nor am I using a different model as Harte repeatedly claims in his comment, but I am computing a different solution of exactly the same MaxEnt problem considered by METE using a different probability distribution. The choice of probability distribution (called Stage 2 in Harte’s comment [3]) seems for him to be a discriminating factor for the solution of a given MaxEnt problem. In my opinion, the choice of the probability distribution should carry no information. The freedom of choice of the probability distribution to describe the information in the system is like the freedom of choice of the reference frame in physics. Once an invariance requirement is assumed and the rules for the change of reference frame are stated, descriptions made in different frames can be compared.

The key point is the following: are we willing to accept that two observers of the same system who are sharing the same initial information on it may obtain nonequivalent probabilistic descriptions of it based on their choice of probability distribution used to set up the MaxEnt procedure? In my opinion, this is unacceptable since it would introduce such a degree of arbitrariness in the inference procedure of MaxEnt to render it useless. As Shore and Johnson expressed in their axiomatic derivation of the MaxEnt principle [4]: if a problem can be solved in more than one way, the results should be consistent.

The theory developed in the first part of my paper addresses precisely this problem. It turns out that, in the application of MaxEnt, the choice of probability distribution *and the form of the entropy function* have to be done accordingly. This is the content of Proposition 1 in [2] which is an elementary result in constrained extremization theory. Simply stated, the solution of a constrained extremization problem does not change if the entropy function and the constraint function are composed with the same diffeomorphism. If the first observer uses *p* and H(p), a second observer using $p^{\prime }$ with $p=g(p^{\prime })$ should use $H^{\prime }\left(p^{\prime }\right)=H\left(g\left(p^{\prime }\right)\right)$ (and the same for the constraint functions). This entails that the use of the entropy function in Shannon form H(p)=−plogp is not always justified. See Section 3 in [2] for an ecologically oriented example.

The natural question that arises is as follows: what choice of probability distribution is justified in using the entropy function in Shannon form? In [2], I suggest determining the form of the entropy function to to be adopted in a given formulation by applying the Boltzmann combinatorial criterion of the most probable distribution (microstate counting). I also show that the entropy function adopted in METE fails to comply with this criterion (tossing individuals into multispecies does not represent independent tosses, see [2]) and therefore in light of the theory developed in [2] it is not the correct entropy function to use. To summmarize, the bivariate distribution R(n,ϵ) used by METE and the one P(n,ϵ) used in [2] are related by a diffeomorphism (Equation (33) in [2]) but the entropy function adopted in METE and [2] are not related by the same diffeomorphism, hence they produce different solutions of the same extremization problem.

## 2. Origin of the Energy–Abundance Correlation in METE

The model considered by METE (see [5,6]) is very simple: a community of $N_{0}$ individuals belonging to $S_{0}$ species and each having a metabolic energy requirement ranging in suitable energy units from 1 to *M*. Let $E_{0}$ be the energy requirement of the whole community. One of the aims of METE is to predict, using MaxEnt, how many species have abundance *n* and how many individuals have energy ϵ. Note that the problem posed resembles closely the Boltzmann problem of the distribution of particles among equally spaced energy levels as well as of species among equally spaced abundance levels. The existence of a negative correlation between the energy and abundance variables in such a simple model, as a result of the application of the MaxEnt procedure, attracted my attention.

To help the reader, consider a non-ecologically oriented model equivalent to the one considered by METE: an urn containing $N_{0}$ billiard balls painted of $S_{0}$ colors (species) and each having a number (the energy requirement) ranging between 1 and *M*. Knowing only $N_{0}$, $S_{0}$ and the sum $E_{0}$ of the ball numbers, would you bet that the numbers and colors are correlated?

For the sake of correctness in the formulation of METE, the initial information in the system is the ratios $N_{0}/S_{0}$ and $E_{0}/S_{0}$ while in my formulation is the ratios $N_{0}/S_{0}$ and $E_{0}/N_{0}$. A second remark raised in Harte’s comment is that these represent a different piece of initial information in the system (Stage 3 in Harte’s comment); therefore, in [2], I am considering a different MaxEnt problem. It is evident that the knowledge of the ratios $N_{0}/S_{0}$ and $E_{0}/N_{0}$ constrains their product which is precisely $E_{0}/S_{0}$. In the two formulations, we are supplying the same initial information in a different form but MaxEnt, as is well known, is able to handle it. To explain it briefly: suppose that one observer considers as initial information the averages a and *b* of two discrete random variables $A=(a_{1},\ldots ,a_{n})$ and $B=(b_{1},\ldots ,b_{n})$ while a second observer uses the averages *a* of *A* and a+b of C=A+B. The MaxEnt solution will be $p_{i}∼\exp\left(-\lambda a_{i}-\mu b_{i}\right)$ for the first and $p_{i}∼\exp\left(-\gamma a_{i}-\beta \left(a_{i}+b_{i}\right)\right)$ for the second. By redefining γ+β=λ and β=μ, we see that the MaxEnt solution is the same. The same argument applies also when considering the ratio of average values.

Now, coming to the issue of the asserted correlation in METE, the author of the comment does not explain the physical causes of this correlation. They are a consequence of the fact that the bivariate distribution R(n,ϵ) is used. It seems implausible to me that, in such a simple model, the sole knowledge of the total number of individuals, the number of species and the total energy requirement induces a (negative) correlation between the way the energy is distributed among individuals and the way the abundance is distributed among species.

Of course more complex models of ecological systems may allow for correlations between energy and abundance. In his comment, Harte cites the population dynamic model by Zhang and Harte in [7] where the resource (energy) allocated to an individual determines its reproductive ability, hence the species abundance. No sort of coupling seems to be acting in METE which is a static model.

In my paper, I have shown in detail that, using a different bivariate probability distribution P(n,ϵ), *the energy and abundance constraints are decoupled* in the sense that they only concern the marginals of the bivariate distribution; hence MaxEnt prescribes, as is well known to its users, a bivariate solution which is the product of two univariate distributions. Moreover, I have shown that if METE structure function R(n,ϵ) is used *but with a different entropy function*, the MaxEnt solution is factorized (Equation (50) in my paper). So, the origin of the correlation found in METE resides in the joint choice of the distribution R(n,ϵ) *and* of the form of the entropy function H(R)=−R(n,ϵ)lnR(n,ϵ) and not in a property of the model describing the ecosystem.

Adopting the theory developed in the first part of [2], one is not tied to a particular form of the probability distribution. To deal with the same initial information considered by METE, I have used two univariate and two bivariate probability distributions and an even METE core structure function R(n,ϵ) and I have always obtained the same MaxEnt solution: the product of two Boltzmann–Gibbs distributions for the energy and abundance variables, which are therefore uncorrelated variables.

## 3. On the Agreement of a MaxEnt Solution with Empirical Evidence

The aim of [2] was not to propose to the scientific community a new form of SAD metric but rather to shed light on a subtle aspect of the application of the MaxEnt procedure which may undermine the construction of a theory. In the application of MaxEnt, the fact that the resulting distribution does not agree with empirical data, is a signal that relevant initial information has been neglected. This is the case for my solution of the METE problem, in agreement with the fact that the billiard ball model at the base of METE is too simple to describe actual ecosystems. On the contrary, the fact that the distribution proposed in METE agrees with empirical data is not proof that the MaxEnt procedure followed there is logically consistent. A series of arbitrarily taken steps may interact to give a widely accepted and empirically confirmed form of SAD, the Fisher log series. As I see it, this is the case of METE. In Harte’s reply, it is said that METE previsions are remarkably accurate for undisturbed ecosystems while they tend to fail for ecosystems subject to anthropogenic disturbances. I am not an ecologist but it seems unlikely to me that such a simple model (equivalent to an urn containing billiard balls) can accommodate information on these fine environmental details. In my opinion, the log series form of the SAD and the existence of a correlation between the abundance and energy requirement in the METE model are a consequence of the form of the probability distribution used and not of the model design.

In the literature dealing with the application of MaxEnt to ecology, it is well known that the sole knowledge of the total number of individuals $N_{0}$ and number of species $S_{0}$ produces an exponential form of the SAD exp(−λn) which is not empirically confirmed. Relevant information in the system has been neglected. A logseries SAD can be recovered if one acknowledges the existence of prior information in the system given by a prior distribution such as 1/n. This prior distribution has been justified by a scale-invariance argument in Pueyo et al. [8] or by considering a dynamical system and the associated master equation (Volkov et al. [9,10]). All of these approaches are beyond the limits of the static model in METE. Another interesting approach, which is relevant to the issues debated here, is contained in the paper [11] by Bowler which I was unaware of when my paper [2] was published. In [11], Section 2.5, the form of the entropy function is derived by a microstate counting using a combinatorial argument different from the one used in Equation (19) of [2]. In particular, it gives rise to a 1/n prior. In the Appendix A, I explain why I disagree with the combinatorial argument used. In Section 2.7 of [11], the author discusses the relevance of his computation for Harte’s METE. Bowler is not entirely correct when he states that individuals in METE are uniquely identified by their species and energy requirement. This is not true because, in METE, many individuals of the same species can have the same energy requirement.

## 4. Conclusions

The first difficulty I encountered when studying METE was the precise understanding of its core object, the bivariate structure function R(n,ϵ) which is defined as follows: the probability that if a species is picked at random from the species list then it has abundance *n*, and if an individual is picked at random from *that species* its metabolic requirement is ϵ (see the paper [5] and Harte’s book [6]).

In [2], I have explained that this definition is logically inconsistent if different species have the same abundance and contain individuals with the same energy requirement, which is likely to be the case. The correct formulation is obtained by substituting “that species” with “a species” or equivalently by saying that the individual has to be chosen from the pool of individuals belonging to species of abundance *n* (called the multispecies *n* in my paper).

I am glad to see that, in his comment, Harte adopts this amended definition and that my disambiguation was useful. I think that a theory has better chances of developing if its core definitions are stated in a precise and logically correct way.

METE (for the part concerning the Species Abundance Distribution) and the solution presented in my paper [2] both claim to be applications of the MaxEnt principle to the same problem but in reality they represent very different procedures from an epistemological point of view. Here, I will try to resume in simple terms these two approaches. In my view (see [2]), MaxEnt is an inference procedure that consists of the following: (1) enumerating the system states; (2) representing the information in the system with a prior distribution and/or by constraints on a chosen distribution; (3) computing the related form of the entropy function from first principles (microstate counting or a set of axioms as in the Shore and Johnson approach). The choice of probability distribution is left to the observer and does not influence the result, in respect to the principle that different observers using the same initial information must obtain the same MaxEnt solution. With a trial and error procedure, new constraints can, in principle, be added until the MaxEnt solution agrees with the empirical data.

In the METE approach, the same system and the same initial information is considered together with a particular choice of probability distribution and of the entropy function without providing the rule to derive the form of the entropy function. It appears that this particular choice produces a solution that agrees with empirical ecological data while others fail to agree. One must conclude that part of the information is not declared as initial information in the system but is contained in the clever choice of probability distribution and the entropy function. However, how this *ad hoc* procedure can be applied to other problems? From this analysis, it seems to me that the path followed in METE is more similar to a successful indirect curve fitting than an application of Maximum Entropy Principle as is intended in literature.

## Appendix A

Let us consider a community of $N_{0}$ individuals belonging to $S_{0}$ species. Following [11], we partition the individuals into classes: class $H_{n}$ (called multispecies $I_{n}$ in my paper [2]) comprises all the individuals belonging to species having abundance *n*, hence it contains $I_{n}=ns_{n}$ individuals where $s_{n}$ is the number of species having *n* individuals. We want to compute the number of ways of ordering the $ns_{n}$ individuals in each $H_{n}$. Due to its definition, it is evident that we can update the content of $H_{n}$ only by adding or subtracting *n* individuals of the same species. Tossing of single individuals in $H_{n}$ does not represent independent tosses as required by the Boltzmann microstate counting procedure. Therefore, in [2], the number of ways is computed as $(I_{n}/n)!$ for each $H_{n}$ giving rise to the combinatorial factor

$$W\left(I\right)=\prod_{n}\left(I_{n}/n\right)!$$

with entropy function $H\left(I\right)=-\sum_{n}\frac{I_{n}}{n}\log\frac{I_{n}}{n}$. In [11], the number of orderings is computed with the following procedure: an individual can be removed from $H_{n}$ in $I_{n}$ ways; then, all the remaining n−1 individuals of the same species are removed. A second individual can be removed in $I_{n}-n$ ways, thus giving rise to the combinatorial factor

$$I_{n}\left(I_{n}-n\right)\left(I_{n}-2n\right)\ldots =\prod_{n}n^{\frac{I_{n}}{n}}\left(I_{n}/n\right)!$$

with entropy function corresponding to the relative entropy with a prior 1/n

$$D\left(\frac{I_{n}}{n}|\frac{1}{n}\right)=\sum_{n}\frac{I_{n}}{n}\log\left(\frac{I_{n}/n}{1/n}\right)$$

Now, I explain why in my opinion the procedure followed in [11] is inconsistent. The number of ways of choosing an object out of *N* is exactly *N* and not N+1 because any additional way would produce a result which has already been seen. In [11], if an individual is chosen and all the remaining n−1 individuals are removed before a second individual is chosen, it means that there are *n* choices of the first individual that produce the same configuration after removing the whole species. Therefore, the genuinely different ways of removing an individual are $I_{n}/n$ and not $I_{n}$.
